# Infrared Thermography for Real-Time Assessment of the Effectiveness of Scoliosis Braces

**DOI:** 10.3390/s23198037

**Published:** 2023-09-22

**Authors:** Leopoldo Angrisani, Egidio De Benedetto, Luigi Duraccio, Fabrizio Lo Regio, Roberto Ruggiero, Annarita Tedesco

**Affiliations:** 1 Department of Electrical Engineering and Information Technology, University of Naples Federico II, 80125 Naples, Italy; angrisan@unina.it (L.A.); fabrizio.loregio@unina.it (F.L.R.); 2 Department of Electronics and Telecommunications, Polytechnic University of Turin, 10129 Turin, Italy; luigi.duraccio@polito.it; 3 Ortopedia Ruggiero SRL, Cardito, 80024 Naples, Italy; info@ortopediaruggiero.it; 4 Department of Chemistry, University of Naples Federico II, 80126 Naples, Italy; annarita.tedesco@unina.it

**Keywords:** Health 4.0, biomedical applications, instrumentation, real-time measurements, real-time monitoring, scoliosis braces, infrared thermal imaging

## Abstract

This work proposes an innovative method, based on the use of low-cost infrared thermography (IRT) instrumentation, to assess in real time the effectiveness of scoliosis braces. Establishing the effectiveness of scoliosis braces means deciding whether the pressure exerted by the brace on the patient’s back is adequate for the intended therapeutic purpose. Traditionally, the evaluation of brace effectiveness relies on empirical, qualitative assessments carried out by orthopedists during routine follow-up examinations. Hence, it heavily depends on the expertise of the orthopedists involved. In the state of the art, the only objective methods used to confirm orthopedists’ opinions are based on the evaluation of how scoliosis progresses over time, often exposing people to ionizing radiation. To address these limitations, the method proposed in this work aims to provide a real-time, objective assessment of the effectiveness of scoliosis braces in a non-harmful way. This is achieved by exploiting the thermoelastic effect and correlating temperature changes on the patient’s back with the mechanical pressure exerted by the braces. A system based on this method is implemented and then validated through an experimental study on 21 patients conducted at an accredited orthopedic center. The experimental results demonstrate a classification accuracy slightly below 70% in discriminating between *adequate* and *inadequate* pressure, which is an encouraging result for further advancement in view of the clinical use of such systems in orthopedic centers.

## 1. Introduction

Scoliosis is defined as a complex deformity of the backbone and the torso that occurs in three dimensions [1,2] and consists of a lateral curvature with a vertebral rotation [3]. The standard screening test for scoliosis is the forward bending test [3], during which the patient is asked to bend forward with straight knees while the examiner observes the back for any signs of asymmetry. If the results of the test, along with the patient’s medical history, raise suspicion of scoliosis, radiography becomes crucial for further evaluation [4]. Once radiography is acquired, scoliosis is identified through the measurement of the Cobb angle, which quantifies the degree of spinal curvature by measuring the angle between the two most inclined vertebrae at the top and bottom of the curve [5,6]. In particular, scoliosis is diagnosed when this angle exceeds 10° [7]. Among the different types of scoliosis, idiopathic scoliosis represents the majority of cases since it is identified as a multi-factor spinal deformity with unknown etiology [8]. In addition to the significant cosmetic deformity, idiopathic scoliosis poses risks including cardiac and pulmonary impairments [9]. Based on the patient’s age, scoliosis is categorized as infantile (0–3 years), juvenile (4–10 years), and adolescent (older than 10 years) [3]. Other classification systems consider the number of curves and the type of deformity [10].

Treatment includes various approaches such as observation, physiotherapy, bracing, and, in extreme cases, surgery [11]. While surgery is needed for Cobb angles greater than 50° [12], scoliosis braces represent the most widely adopted treatment for patients with incomplete bone growth and Cobb angles ranging between 25° and 50° [4,12]. In this particular scenario, patients wear a rigid or semi-rigid corset-like device, whose model differs in *Milwaukee*, *Lyonnaise*, *Cheneau*, *Sforzesco*, *Boston*, and others [4], based on the patient’s bone maturity, Cobb angle, and backbone deformation [4]. The design of this corset is tailored to suit the individual patient’s torso, considering the asymmetry caused by scoliosis, while the primary objective is to realign the patient and correct the curvature of the backbone. To achieve this, the corset applies external pressure specifically to the regions of the backbone that are affected by the curvature.

During treatment, regular follow-up examinations are necessary to evaluate brace compliance and adjust the corset according to the changes in the patient’s body [13], ensuring proper pressure application. However, currently, there is no consensus in the literature on the implementation of these brace corrections [12], and there is also a lack of agreement on the mechanical principles of brace design and manufacturing [8,14]. As a result, the evaluation of the effectiveness of the brace, that is deciding whether the pressure exerted by the brace is considered *adequate* or *inadequate*, relies entirely on the expertise of the orthopedist [2,15]. Hence, a more reliable measure to confirm the orthopedist’s opinion is the assessment of curve progression, typically achieved by comparing the Cobb angle measured through radiographic images taken over a specific period of time [16].

As can be deduced, this approach requires a certain time interval between the two measurements of the Cobb angle. In addition, when using radiographic imaging, the potential risks associated with ionizing radiation exposure constitute a limitation for repeated acquisitions over time. If alternative radiation-free methods, such as Moiré topography [17] or 3D scanning [18], are employed to assess the curve progression and evaluate the effectiveness of the brace, the time horizon between the two acquisitions could be considerably shortened. Nevertheless, immediate evaluation remains infeasible, as a gradual reduction in the spinal curvature can only be achieved with the prolonged wearing of the brace by the patient. Moreover, another crucial aspect is that failure to wear the corset correctly by the patient could result in a deterioration of scoliosis, even if the corset has been properly designed. Therefore, a comparison of two measurements over time may not accurately reflect the effectiveness of the corset if it is not consistently and correctly worn as prescribed. Consequently, orthopedists still currently lack an objective means of monitoring the effectiveness of corsets in real time, which would enable prompt adjustments to be made.

A first attempt at enabling real-time evaluation was introduced in [10], where the considered technique involved the monitoring of the mechanical pressure exerted by the brace using pressure sensors positioned between the brace and the patient’s backbone. Nevertheless, measuring the pressure between these two surfaces while consistently moving the sensor, without compromising the accuracy of the measurement, proved to be a challenging task. Therefore, ensuring reliability, repeatability, and cost-effectiveness for widespread implementation in healthcare facilities posed additional complexities.

Starting from these considerations, this study presents an innovative, non-invasive, and cost-effective approach for evaluating the effectiveness of scoliosis braces in real time. The proposed method utilizes low-cost infrared thermography (IRT) instrumentation to acquire the skin temperature of the patient’s back, immediately after removing the braces.

By processing the acquired temperature data, the developed system can determine whether the mechanical pressure applied by the corset was *adequate* or *inadequate* according to the orthopedic prescription and design of the brace. In practical applications, this method can provide orthopedists with a reliable and objective assessment, allowing them to promptly identify the need for adjustments to the corset and enhance the scoliosis treatment process. This could represent a possible alternative to reduce the prescription of X-rays.

This paper is organized as follows. Section 2 provides background on IRT technology, with a focus on relevant application scenarios in healthcare. Section 3 describes the proposed method. The experimental validation is reported in Section 4, along with the obtained results. Finally, in Section 5, some conclusions are drawn and future works are outlined.

## 2. Background

IRT is a non-invasive technology that relies on the detection and registration of emitted radiation energy at wavelengths ranging from 2 to 15 μm [19]. This is achieved through an array of detectors that convert the energy *E* into a thermal image [20] that displays the temperature *T* of the observed objects as per the Stefan–Boltzmann law $E=\varepsilon \;\sigma \;T^{4}$, where ε represents the emissivity of the objects, which is defined as the ratio between the amount of infrared energy emitted by the object and that emitted by an ideal black body at the same wavelength and temperature [21], and σ is the Stefan–Boltzmann constant.

The amount of energy emitted by an object is influenced by multiple factors, including not only emissivity but also wavelength and surface temperature. As emissivity values vary among different objects, they can emit the same amount of thermal energy, even at different temperatures. Moreover, when utilizing infrared detectors to measure the infrared energy emitted by a specific object, the measured value may not solely reflect the energy emitted by the object itself. As a matter of fact, it is also influenced by the energy absorbed, reflected, and emitted by the surrounding environment [20]. In addition, the measure also depends on the distance between the surface and the camera [22].

**Figure 1:**
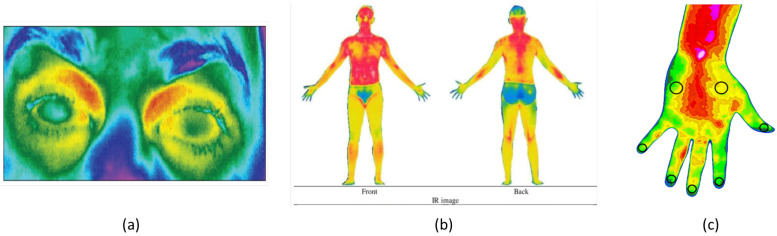
Examples of adoption of IRT in the frameworks of (**a**) the evaluation of ocular inflammation [36], (**b**) ergonomic assessment [41], and (**c**) diagnosis of carpal tunnel syndrome [38].

IRT technology has experienced widespread adoption across diverse fields, including electrical engineering [23], mechanical engineering [24], agriculture [25], veterinary medicine [26], and healthcare [27]. With regard to the healthcare sector, this technology has made significant strides over the years, benefiting from advancements in detector sensitivity, cost reductions [22,28], and suitable integration within the broader context of the *4.0* digital transition, which leverages enabling technologies like Augmented Reality [29], the Internet of Things [30], Cloud Computing [31], and Artificial Intelligence [32,33]. As a matter of fact, these advancements have resulted in the development of attached-to-smartphone infrared cameras, which offer improved portability, connectivity, and ease of use, without compromising performance, compared to traditional devices [34]. This has paved the way for the rise of decision-support systems that can furnish healthcare professionals with fast, reliable, and objective results in diverse scenarios, including the evaluation of inflammatory processes [35,36], detection of infections, [37] diagnosis of carpal tunnel syndrome [38], monitoring of diabetes-related conditions [39], and assessment of eye diseases [40]. In the field of rehabilitation and orthopedics, these systems are used for ergonomic evaluations [41], injury prevention and assessment [42,43], scoliosis diagnosis [44,45], and brace manufacturing [46]. In Figure 1, some of the aforementioned healthcare-related scenarios are illustrated.

All these scenarios require advanced knowledge of the relationship between the human body and the relative emitted thermal energy. Human skin has a constant emissivity in the range of 3–15 μm of about 0.97±0.05, close to that of the black body [22], while the contribution to the heat supply emitted by the human body can be mainly related to blood perfusion, metabolism, and external sources [27,47] such as electromagnetic fields or mechanical loading [27]. In the latter case, the relationship between mechanical loading and emitted thermal energy allows the use of IRT to evaluate the stress imposed on a body. The analysis involved is known as Thermoelastic Stress Analysis (TSA) and it is based on the thermoelastic effect, which refers to the linear relationship between changes in body temperature (and thus emitted thermal energy) and stress states on the surface of the body, assuming local adiabatic conditions [27]. In more detail, mechanical loading is related to the skin temperature variations on a patient’s back according to (1) [48]:

(1) $$\Delta T=\frac{T}{\rho \;C_{\varepsilon }}\sum \frac{\partial \sigma_{ij}}{\partial T}\varepsilon_{ij}+\frac{Q}{\rho \;C_{\varepsilon }}\;$$

where *T* is the absolute temperature of the body, $C_{\varepsilon }$ is the specific heat at a constant strain, ρ is the density, *Q* is the heat input, and $\sigma_{ij}$ and $\epsilon_{ij}$ are, respectively, the stress and strain change tensors in the three dimensions for *i*, *j* = {1, 2, 3}.

When taking all these factors into account, it becomes clear that in the framework of the evaluation of the effectiveness of scoliosis corsets, TSA could represent a robust foundation that can be exploited to assess, through suitable acquisition and processing of the thermal images of a patient’s back, whether the pressure applied by the corset is adequate.

## 3. Proposal

**Figure 2:**
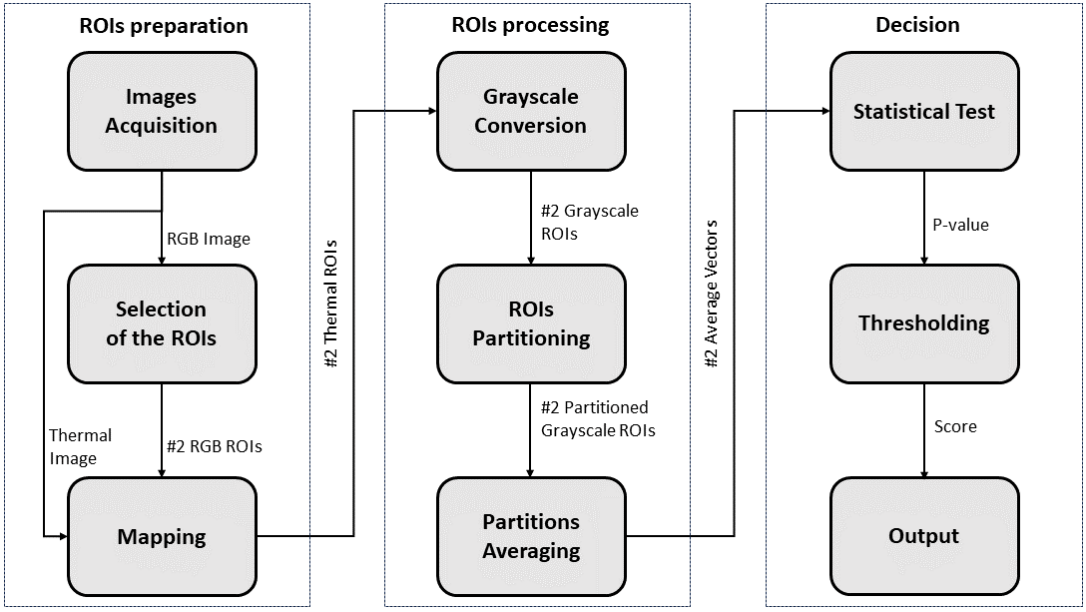
Conceptual description of the proposed method.

Based on the considerations outlined in Section 1 and Section 2, this study proposes a method that leverages the relationship between skin temperature variations and applied mechanical pressure to evaluate whether the pressure applied by a scoliosis corset on a patient’s back is *adequate* or *inadequate*, thus facilitating an orthopedist’s clinical decision making. The proposed method represents a *workaround* to the problem of directly measuring the pressure exerted by the brace, which is a task associated with several difficulties, as reported in [10]. Figure 2 schematizes the pipeline of the method, which consists of three major modules, namely *Regions of Interest (ROIs) preparation*, *ROIs processing*, and *Decision*.

1. The *ROIs preparation* module consists of three blocks.
  The first block, named *Images Acquisition*, captures the thermal and corresponding RGB images from the dorsum of the patient, immediately after removing the brace. It is noteworthy that the patient’s dorsum remains uncovered during this stage. To ensure that the bracing effect remains visible, it is recommended to wait no more than one minute between the patient removing the scoliosis corset and the start of image capturing. In fact, the duration of the corset’s pressure effect on skin temperature variation after its removal can be influenced by several factors, such as the duration of brace usage, the intensity of the applied pressure, the patient’s metabolism, sweating, and the ambient temperature. This effect may gradually dissipate within a few minutes or persist for an extended period ranging from several minutes to tens of minutes [49,50]. Hence, a waiting time of less than one minute can be considered a time to ensure adequate stability in the short term.
  In the second block, referred to as the *Selection of the ROIs*, the orthopedic specialist selects on his/her computer (with the help of cursors) two ROIs on the acquired RGB image: the first ROI corresponds to the area in which the thrust is exerted by the brace, whereas the second ROI is selected symmetrically to the first ROI with respect to the backbone. It should be pointed out that this selection is guided by the patient’s clinical history: the orthopedic specialist has access to the patient’s radiography, has knowledge of the diagnosis, knows the type of corset worn, and has the related prescription. As a result, he/she possesses the necessary information to identify the specific region of the back where the corset needs to exert its effect. Nevertheless, to avoid confirmation bias, the selection of the ROIs is not performed directly on the thermal image but rather on the RGB one.
  Finally, the third block (*Mapping*) is responsible for mapping the selected regions from the RGB image onto the thermal image.
2. The *ROIs processing* module is divided into three blocks.
  The first block, named *Grayscale Conversion*, handles the conversion of the thermal ROIs from the RGB color space to grayscale, where white is associated with the maximum temperature value and black is associated with the minimum temperature value. Consequently, each ROI undergoes a transformation from three dimensions (red, green, and blue channels) to one dimension (grayscale) to save computational effort.
  Then, in the *ROIs Partitioning* block, each ROI converted to grayscale is divided by performing both horizontal and vertical slicing. As a result, each ROI is segmented into N×M subregions, where *N* represents the number of horizontal slices and *M* represents the number of vertical slices.
  In this way, the last block, called *Partitions Averaging*, performs an average assessment on each of the N×M subregions within the partitioned grayscale ROIs. This process generates two vectors, each with dimensions [N×M, 1], corresponding to the averaged values of the temperature of each ROI subregion.
3. These two vectors are compared through the *Decision* module.
  In particular, a *Statistical Test* is performed between the two vectors to evaluate whether there is a statistically significant difference between the means of the two groups represented by the vectors. The output of this test is the *p*-value, which indicates the probability of obtaining test results at least as extreme as the result actually observed, under the assumption that the null hypothesis is correct. In this context, the null hypothesis implies no significant difference between the two vectors, suggesting inadequate scoliosis brace pressure. For this reason, the lower the *p*-value, the lower the probability of erroneously rejecting the null hypothesis. The utilization of a statistically derived score affords independence from absolute temperature (and consequently, pressure) values measured on the patient’s back, which significantly vary among different patients and corsets, given the anatomical distinctions inherent to each individual. As a matter of fact, typical pressure values range from 7 to 10 kPa [51], but these values are subject to significant variability, both inter-subject and intra-subject.
  The resulting *p*-value is compared with a *Threshold* to associate it with an *Output* that can indicate whether the scoliosis brace is functioning adequately. More specifically, if the obtained *p*-value is found to be lower than the threshold value, and if the average temperature of ROI #1 (region where brace pressure is assumed to be) is greater than that of ROI #2 (region where brace pressure is not assumed to be), the pressure of the scoliosis corset is indicated as adequate. Conversely, if the *p*-value exceeds the threshold value, it is indicated as inadequate. The identification of this threshold can follow an a priori model, which is based on prior information, or models based on learning from newly acquired data.

**Figure 3:**
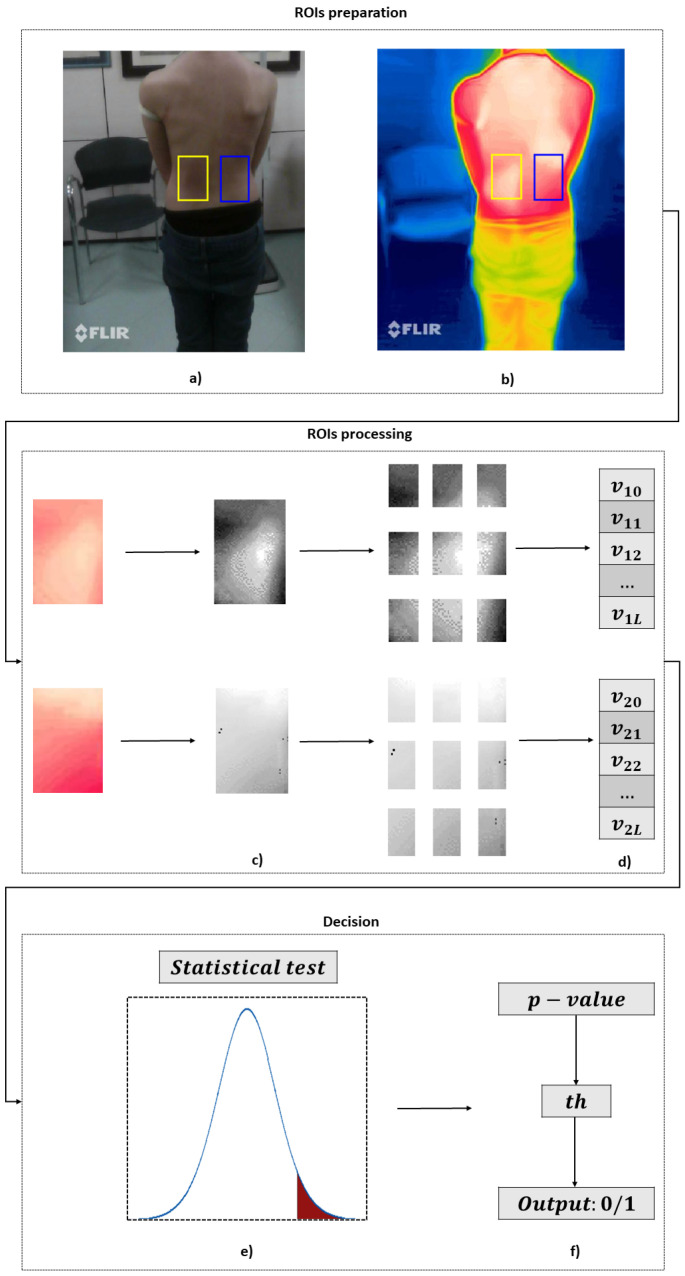
Graphical representation of the proposed method. (**a**) Selection of the ROIs. (**b**) Mapping. (**c**) ROIs partitioning and grayscale conversion. (**d**) Partition averaging. (**e**) Statistical test. (**f**) Thresholding and output.

A graphical representation of the proposed method is shown in Figure 3. The three modules (*ROIs preparation*, *ROIs processing*, and *Decision*) are highlighted, along with the inner blocks related to the selection of the ROIs (a); the mapping onto the thermal image (b); the partitioning and grayscale conversion (c); the averaging of the partitions (d), which provides two vectors *v* of length L=N×M; the *t*-test (e); and, finally, the thresholding and output assessment (f), which is 0 if the corset pressure is inadequate and 1 otherwise.

## 4. Experimental Validation

This section describes the experimental validation of a system developed based on the proposed IRT-based method. First, the experimental setup is described, along with the experimental study conducted on patients. Then, the performance of the developed system is evaluated using suitable validation strategies.

### 4.1. Experimental Setup

**Figure 4:**
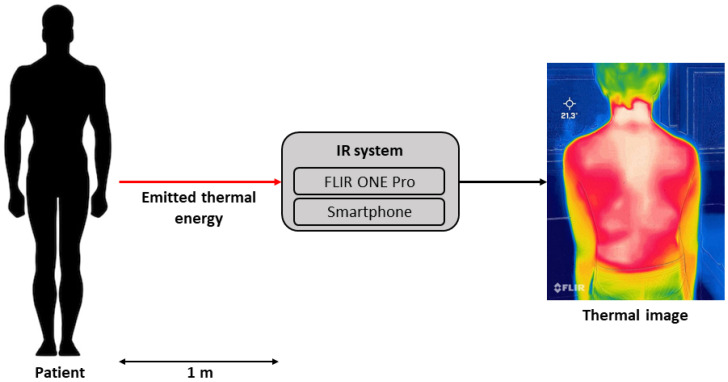
Sketch of the acquisition system.

The acquisition of the thermal images was performed using the *FLIR ONE Pro* thermal imaging camera [52], a low-cost attached-to-smartphone camera. The cost of this camera is approximately USD 450. In terms of metrological performance, the camera provides an accuracy of 3 °C when operated within a temperature range of 15 to 35 °C and when measuring object temperatures ranging from 0 to 120 °C. The thermal sensitivity is equal to 100 mK. The thermal sensor of the camera operates within a spectral range of 8 to 14 μm, encompassing the range of interest from 8 to 12 μm. The acquired data are stored directly on the smartphone as images with dimensions of 1440 × 1080 pixels, while the thermal resolution of the camera is 160 × 120 pixels. In accordance with the methodology outlined in [41,42], each patient was positioned at a specified distance from the camera. A marked spot on the floor, situated 1 m away from the IR camera, was designated as the reference point. This approach was employed to ensure the repeatability and reproducibility of measurements, as it allowed us to guarantee the same camera’s performance in terms of resolution and minimized interference from objects near the patients throughout the entire study. All possible obstacles between the IR camera and the patient’s back were carefully avoided. For the sake of completeness, a sketch of the acquisition system is shown in Figure 4.

### 4.2. Experimental Study

**Figure 5:**
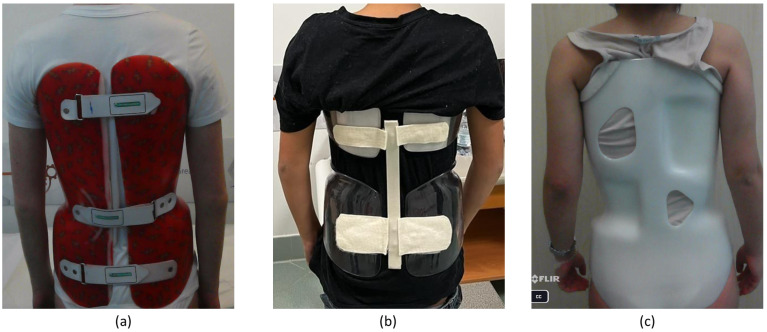
Examples of different brace models, (**a**) *Boston*, (**b**) *Sforzesco*, and (**c**) *Cheneau*, worn by three subjects involved in the experimental study.

The experimental study was conducted at the *Ortopedia Ruggiero* site in Cardito (Naples, Italy) and included a cohort of 21 patients categorized as juvenile and adolescent, of which fourteen were females. This patient distribution reflects the evidence that idiopathic scoliosis is more prevalent in women [53]. All the patients were affected by idiopathic scoliosis and subjected to bracing treatment; hence they were not under consideration for surgery and wore different braces according to the specialist’s prescription, as shown in Figure 5. Six patients were affected by dorsal or lumbar scoliosis, and the remaining fifteen suffered from dorso-lumbar scoliosis with a double curve of the backbone. Furthermore, no patient was affected by chronic or acute health conditions that would cause temperature changes in the skin’s surface. Overall, nineteen patients participated in the experimentation once, whereas two patients were acquired twice during the course of the study.

Before the IR acquisition, the patients were asked to avoid stimulant beverages, physical activity, body creams, and wearing jewelry. The experimental study was carried out in an air-conditioned room with non-direct airflow at the patients and a temperature ranging from 19 °C to 23 °C, which is representative of real operating conditions.

Upon patients’ arrival at the facility, their radiographs and orthopedist prescriptions were obtained. Subsequently, they were instructed to rest in a designated room for approximately fifteen minutes to acclimate. During this time, the orthopedist conducted a standard examination of the patient, including an assessment of the brace’s compliance based on manual procedures.

After acclimatization, patients were instructed to undress and remove the brace, allowing for thermal images of their back to be captured. This step ensured that any obstruction caused by the brace material was eliminated, enabling clear visualization of the thermal effects resulting from the brace’s applied pressure.

At the end of the experimental study, a total of 21 pairs of RGB/thermal images were obtained (one for each patient). For each RGB image, the medical team selected the ROIs, as described in Section 3. With regard to the patients who suffered from scoliosis with a single curve of the backbone, only one pair of ROIs was selected. However, for those suffering from scoliosis with a double curve, two pairs of ROIs were selected.

Overall, the medical team selected 36 pairs of ROIs and assigned a label $Y_{i}$ to each of them to indicate the pressure applied by the brace as adequate/1 or *inadequate/0*. The labeling process followed a majority rule to minimize subjective evaluations from a single operator, thus avoiding bias.

### 4.3. Performance Evaluation

The acquired 36 pairs of ROIs were processed in the MATLAB environment, as described in the *ROIs processing* module shown in Figure 2. After confirming that the data belonged to a normal distribution (through an $\chi^{2}$ test), the statistical test chosen to provide the scores associated with each pair of ROIS was the Student’s *t*-test. Therefore, the dataset to be analyzed was composed of 36 scores $X_{i}$, each one associated with the label $Y_{i}$. To evaluate the performance of the developed system in terms of classification accuracy (defined as the percentage of the instances of *X* correctly classified) and generalization capability (overfitting prevention), a leave-one-out cross-validation (LOOCV) strategy was applied.

LOOCV is a common method used to assess the performance and generalization ability of a classifier in a dataset; it is a form of k-fold cross-validation, where *k* is equal to the number of instances in the dataset. In LOOCV, the dataset is divided into *k* subsets or folds, where each fold contains only one instance. The model is trained on k−1 folds and then tested on the remaining fold. This process is repeated *k* times, with each instance serving as the test set once.

In this study, the training was performed by leveraging a grid search between 1000 different values of the threshold th, ranging from 0.005 to 0.500. For each iteration, the threshold value $th_{max}$ that maximized the classification accuracy on the k−1 training folds was used on the test fold *k*. At the end of the LOOCV process, the classification accuracies obtained from each iteration (defined as the percentage of instances correctly classified) were averaged to obtain a final evaluation of the model’s performance. This average performance serves as an estimate of how well the model is likely to perform on unseen data.

**Figure 6:**
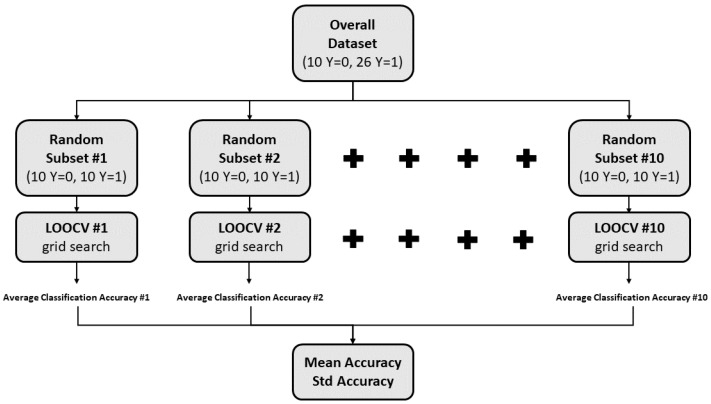
Description of the evaluation of system performance.

However, due to the significant class imbalance in the dataset, with only 10 instances labeled as *inadequate/0* pressure and 26 instances labeled as *adequate/1* pressure, a balancing procedure was conducted prior to the application of leave-one-out cross-validation (LOOCV). Specifically, ten random subsets were created from the original dataset, ensuring that each subset consisted of 20 balanced instances, with half of them labeled as 0 and the remaining half labeled as 1. The procedure for creating each of the ten random subsets was based on randomly selecting 10 instances of the dataset out of the 26 labeled as *1* (changing the seed each time), to which the 10 instances of the dataset labeled as *0* were added. Therefore, LOOCV was applied for each of the ten subsets, thus obtaining ten different values of averaged classification accuracy and related standard uncertainty (evaluated as type-A uncertainty [54]). In this way, the overall mean value and uncertainty extracted provide a robust indication of the system performance on unseen data. Figure 6 provides an illustration of the evaluation of the system performance.

The accuracy *A* and the corresponding standard uncertainty *u*, obtained for each subset and then averaged, are shown in Table 1, expressed as percentages.

As can be seen, the overall mean accuracy $A_{m}$ was equal to 65.5%, whereas the overall standard uncertainty $u_{m}$, evaluated using the first-order law of the propagation of uncertainty [54], was found to be equal to 3.4%. Assuming a normal distribution and a confidence interval of 95%, a coverage factor k=2 was applied to obtain the expanded uncertainty $U_{m}=k\cdot u_{m}$ and express the measurement results as (65.5±6.8)%.

Taking into consideration the employed instrumentation and the approach used in the experimental study, intentionally designed to simulate real-case scenarios, this result proves to be promising regarding further enhancements and potential clinical applications of the system in orthopedic centers. By utilizing such a system to assess the effectiveness of scoliosis braces, orthopedic specialists would have objective support that can significantly contribute to the decision-making process. This could lead to further enhancements in the practice of brace-based treatments, eliminating the need to solely rely on the evaluation of curve progression over time before making decisions regarding necessary brace adjustments.

## 5. Conclusions

This work proposed a method based on low-cost infrared thermography instrumentation for the real-time evaluation of the effectiveness of scoliosis braces. The proposed method leverages the thermoelastic effect to correlate changes in brace pressure with temperature variations on the patient’s back. An experimental study at an accredited orthopedic center was conducted on 21 patients of juvenile and adolescent age, simulating real operational conditions and acquiring 36 regions of interest, each of which was labeled by the medical team. A dedicated algorithm incorporating a typical machine learning validation technique was implemented to ensure generalization to unseen data. The experimental results demonstrated a classification accuracy of slightly below 70%, which represents a promising value considering the use of low-cost instrumentation and intentionally non-ideal experimental conditions.

This study represents a pioneering effort in utilizing systems based on this method for clinical applications. By employing such systems to assess the effectiveness of scoliosis braces, orthopedic specialists can have objective support that significantly contributes to the decision-making process. These findings have the potential to drive further advancements in brace-based treatments, reducing the sole reliance on evaluating curve progression over time before making brace adjustments. Future research will focus on enhancing performance through the implementation of more advanced instrumentation, gathering additional data such as temperature decay curves when patients remove their braces, improving the ROI selection (eventually using marker-based approaches), and adopting more sophisticated algorithms to enhance the reliability of this method for orthopedic centers. This also paves the way for new evaluations of the effectiveness of the therapy, based on the observation of the compensation of asymmetric skin temperature distribution along the paravertebral areas over time.

## Figures and Tables

**Table 1:** Accuracy (A) and corresponding standard uncertainty (u) obtained for each subset and then averaged.

Metric	Set #1	Set #2	Set #3	Set #4	Set #5	Set #6	Set #7	Set #8	Set #9	Set #10	Mean
*A* (%)	70.0	55.0	65.0	75.0	65.0	70.0	60.0	65.0	65.0	65.0	**65.5**
*u* (%)	10.5	11.4	10.9	9.9	10.9	10.5	11.2	10.9	10.9	10.9	**3.4**

## Data Availability

Not Applicable.
